# Impaired autonomic function and somatosensory disturbance in patients with treated autoimmune thyroiditis

**DOI:** 10.1038/s41598-024-63158-w

**Published:** 2024-05-29

**Authors:** Bojana Bazika-Gerasch, Nina Kumowski, Elena Enax-Krumova, Miriam Kaisler, Lynn Bernadette Eitner, Christoph Maier, Johannes W. Dietrich

**Affiliations:** 1 Diabetes, Endocrinology and Metabolism Section, Department of Internal Medicine I, St. Josef Hospital, Ruhr University Bochum, Gudrunstr. 56, 44791 Bochum, NRW Germany; 2 Diabetes Centre Bochum/Hattingen, St. Elisabeth-Hospital Blankenstein, Im Vogelsang 5–11, 45527 Hattingen, NRW Germany; 3 Centre for Diabetes Technology, Catholic Hospitals Bochum, Ruhr University Bochum, NRW, Gudrunstr. 56, 44791 Bochum, Germany; 4 Department of Internal Medicine 1, University Hospital Aachen, Rheinisch-Westfälische Technische Hochschule (RWTH) Aachen University, Pauwelsstraße 30, 52074 Aachen, NRW Germany; 5 Department of Neurology, BG University Hospital Bergmannsheil gGmbH, Ruhr-University Bochum, Bürkle-de-la-Camp-Platz 1, 44789 Bochum, NRW Germany; 6 Pediatrics Department, Catholic Hospitals Bochum, Ruhr University Bochum, Alexandrinenstraße 5, 44791 Bochum, NRW Germany; 7 Centre for Rare Endocrine Diseases, Ruhr Centre for Rare Diseases (CeSER), Ruhr University Bochum and Witten/Herdecke University, Alexandrinenstr. 5, 44791 Bochum, NRW Germany; 8 Centre for Thyroid Medicine KKB, Catholic Hospitals Bochum, Ruhr University Bochum, Gudrunstr. 56, 44791 Bochum, NRW Germany

**Keywords:** Hypothyroidism, Autoimmune thyroiditis, Autonomic neuropathy, Heart rate variability, Quantitative sensory testing, Somatosensory function, Thyroid diseases, Neuropathic pain, Peripheral neuropathies

## Abstract

Despite treatment with levothyroxine, hypothyroidism and autoimmune thyroiditis (AIT) may be associated with reduced quality of life (QoL), an enigmatic condition referred to as "syndrome T". Peripheral neuropathy, described in untreated thyroid disease, could be a contributing mechanism. We analysed autonomic and somatosensory function in 29 patients with AIT and treated hypothyroidism and 27 healthy volunteers. They underwent heart rate variability (HRV) analysis and quantitative sensory testing (n = 28), comprising 13 parameters of small and large nerve fibre function and pain thresholds. Autonomic cardiovascular function was assessed in rest, deep respiration and orthostasis. Additionally, biomarkers for autoimmunity and thyroid function were measured. Anxiety, depression and QoL were assessed using validated questionnaires. 36% of the patients showed at least one sign of somatosensory small or large fibre dysfunction. 57% presented with mild hyperalgesia to at least one stimulus. Several markers of autonomic function and some detection thresholds were related to the antibody titres. Anxiety, depression scores and QoL correlated to antibody titres and HRV measures. Autonomic and somatosensory dysfunction indicate that in treated hypothyroidism and AIT a subgroup of patients suffers from neuropathic symptoms leading to impaired QoL. Additionally, mild hyperalgesia as a possible sensitisation phenomenon should be considered a target for symptomatic treatment.

## Introduction

A considerable proportion of subjects affected by hypothyroidism suffer from impaired psychological well-being, reduced quality of life, autonomic symptoms and diffuse pain, even if treated with levothyroxine and if normal TSH indicates sufficient substitution^1–7^. This enigmatic condition, also referred to as “syndrome T”^5,8^ or “syndrome of residual symptoms of hypothyroidism on T4” (SORSHOT)^9^ affects about 0.5% of the inhabitants of developed countries^2^. A plethora of possible underlying mechanisms have been suggested, including psychological factors, the presence of other autoimmunity diseases, thyroid autoimmunity, insufficient dosage, hysteresis effects, the modality of therapy with levothyroxine (potentially resulting in abnormal T3 generation) and polymorphic variants of deiodinase genes^2,10–12^.

The possible presence of peripheral neuropathy in hypothyroidism is currently underappreciated, although it might explain parts of the clinical spectrum. There is a large body of evidence for sensorimotor polyneuropathy and other peripheral neuropathies in patients with untreated hypothyroidism, as demonstrated by diverse methods including sensory and motor nerve conduction velocity, handheld dynamometry, electromyography, quantitative sensory testing and neurological examination^3,13,14^. Reduced intraepidermal nerve fibre density was observed in both overt and subclinical hypothyroidism^15^. Furthermore, in overt hypothyroidism the central nervous system is commonly affected as well^16^. Several studies described peripheral nerve abnormalities in hypothyroidism as reversible after initiation of substitution therapy with levothyroxine^3,17,18^.

However, the extent and impact of altered autonomic and somatosensory function on clinical complaints in patients with treated hypothyroidism remains unclear. In patients with treated hypothyroidism and symptoms and signs of polyneuropathy, one study found a high proportion of peripheral nerve abnormalities, including nerve conduction studies and reduced intraepidermal nerve fibre density, as well as abnormalities in quantitative sensory testing (QST) overall. However, in nearly the half of the included patients, also possible other causes for polyneuropathy could be identified^4^.

Orthostatic hypotension has been described in cases of hypothyroidism^19^, but the prevalence is unclear in the absence of concomitant diabetes mellitus, systemic rheumatic diseases and adrenal failure, and the mechanisms remain to be poorly understood.

The present study had the aim to examine whether patients with treated hypothyroidism display signs of altered somatosensory function in quantitative sensory testing (QST). Additionally, we aimed at investigating the function of the cardiovascular autonomic nervous system in the included subjects. A third question to be addressed was if hormone concentrations and thyroid antibody titres correlate to markers of somatosensory or autonomic nerve function and if abnormal thyroid function was common in affected subjects. Not least we wanted to investigate the association of these parameters with the quality of life.

## Methods

### Subjects

This study was part of the RUBIONERVE platform, a large research project on mechanisms and phenotypical patterns of neuropathy in different pathophysiological conditions^20^. It included subjects with autoimmune thyroiditis (AIT) and a control group of healthy volunteers. The patients were recruited from the Department of Endocrinology, Bergmannsheil University Hospitals in Bochum, NRW, Germany. Healthy subjects were recruited among the staff of the hospital and via personal invitation.

Inclusion criteria for patients were AIT, as evidenced by elevated titres against thyroglobulin or thyroid peroxidase in the present or past, or a hypoechogenic and inhomogeneous pattern in thyroid ultrasound^21,22^, and age of 18 years or older. Inclusion criteria for healthy subjects were the absence of any known thyroid disease and an age of 18 years or older. Exclusion criteria for both groups were severe CNS affections, other reasons involving neuropathy, e.g. diabetes, treatment with platin-based chemotherapy and systemic rheumatic diseases, substitutive treatment with L-T3 or TRIAC, Graves' disease, Riedel's thyroiditis, pituitary diseases, current major illness potentially leading to NTIS/TACITUS and pregnancy.

The healthy control group was matched in age and gender to the AIT group. The selection process followed the recommendations of a consensus statement by the EUROPAIN and NEUROPAIN consortia for including healthy volunteers in QST studies^23^.

The study was performed after approval by the ethics committee of the medical faculty of the Ruhr-University Bochum was received (registration no. 4905–14). Written informed consent was obtained from all patients and healthy subjects after a thorough explanation of the aims of the study and necessary examinations according to the latest version of the Declaration of Helsinki.

### Laboratory tests

All patients underwent blood collection for concentrations of thyroid-stimulating hormone (TSH), free triiodothyronine (FT3), free thyroxine (FT4) and antibody titers, including anti-thyroid peroxidase antibodies (TPOAb), anti-thyroglobulin antibodies (TgAb) and TSH receptor antibodies (TRAb). Concentrations of TSH, FT4, and FT3 were determined with a fully automated chemiluminescence-based system (DxI800, Beckman-Coulter, Krefeld, Germany). The intra-assay and inter-assay CVs for these analyses vary with concentrations but are < 10% for the range of measurement^24^. Thyroid tissue antibodies (TPOAb, TgAb, and TRAb) were measured with quantitative radioimmunoassays (anti-TPOn, anti-Tgn and TRAKhuman, ThermoFisher, BRAHMS division, Henningsdorf, BB, Germany).

To assess the relative contributions of the pituitary gland, the thyroid and peripheral tissues to the variations in hormone concentrations, Jostel’s TSH index (JTI), the thyroid’s secretory capacity (SPINA-GT) and the sum activity of peripheral deiodinases (SPINA-GD) were calculated from steady-state concentrations of TSH, thyroid hormones and constants for plasma protein binding and kinetics, as recently described and recommended for thyroid trial design^24–26^. SPINA-GT was only calculated in subjects not on levothyroxine substitution therapy. The equations used are provided along with the required parameters in the supplementary material. Local reference ranges were 0.35–3.5 mIU/L for TSH, 3.5–6.3 pmol/L for FT3, 7.7–18 pmol/L for FT4 and < 60 IU/ml for anti-TPO antibodies, < 60 IU/ml for TgAb and < 2 IU/ml for TRAb. Anti-TPO antibody titres between 60 and 1000 IU/ml were classified as intermediate and titres higher than 1000 IU/ml as high^27,28^.

### Thyroid ultrasound investigation

All subjects underwent an ultrasound investigation of the thyroid gland that was performed by a single experienced investigator. The investigation followed recommendations by the World Health Organisation and the Iodine Global Network. The thyroid volume was calculated with Brunn's equation as

$$V = length \cdot width \cdot depth \cdot 0.479$$

for each lobe^29^. Echogenicity pattern and perfusion were recorded for the parenchyma in both lobes and the isthmus.

### Questionnaires

Quality of life and symptoms of anxiety and depression were assessed with the SF-36 questionnaire and the hospital anxiety and depression scale (HADS), respectively.

### Testing for cardiovascular autonomic function

The autonomic function was investigated by measuring the heart rate variability (HRV) in resting state and under defined test conditions (deep respiration and Ewing's orthostasis test). Obtained ECG recordings were analysed with a computer-based system (ProSciCard III®, software version 2.5, MEWICON CATEEM-Tec GmbH, D-94089 Neureichenau, Germany). The ECG amplifier had a sampling rate of 1400 Hz and was connected to the computer with a photocoupler to provide galvanic isolation. The software provided for semi-automatic outlier and artefact detection. Artefacts were removed before analysis, and recordings with a high number of artefacts were obtained a second time. Preparation of volunteers and rooms and data acquisition followed current recommendations for procedures of HRV analysis^30^. Data analysis, comprising measures in the time and frequency domain, followed the recommendations of the Task Force of the European Society of Cardiology and the North American Society of Pacing Electrophysiology^31^ and the German Diabetes Society^32^. The following time domain-based HRV indices were used for analysis: SD NN and CoV NN (standard deviation and coefficient of variation of normal artefact-free RR intervals), RMSSD (root mean square of successive differences), MCR (mean circular resultant), HRV triangular index (Integral of the density of the RR interval histogram divided by its height), TINN (baseline width of the RR interval histogram), average R-R_max_–R-R_min_ and average R-R_max_/R-R_min_ (representing the longest and shortest R-R intervals in the breath cycle)^33^. In the frequency domain, spectral power density was computed in three frequency ranges (VLF: 0.003–0.04 Hz, LF: 0.04–0.15 Hz and HF: 0.15–0.4 Hz, measured in ms^2^), and from the results, Gerritsen’s ratio^34^ was calculated as

$${R}_{G}=\frac{LF}{LF+HF} .$$

Additionally, the drop in blood pressure in Ewing’s orthostasis test was recorded. The difference in blood pressure was defined as the lowest value in repeated measurements during a five-minute follow-up minus the last value before transition to orthostasis.

### Quantitative sensory testing

All QST examinations were performed under standardized conditions in a certified QST laboratory following the protocol of the German Network on Neuropathic Pain (DFNS) to ensure a high quality of the QST^35^. The DFNS protocol is described in detail by Rolke et al.^36^. In short, QST comprises 13 parameters of small and large nerve fibre function, including the corresponding central pathways, as well as sensitization processes. It assesses thermal detection for cold and warm stimuli (CDT and WDT: cold and warm detection threshold), pain thresholds for cold and hot stimuli (CPT and HPT: cold and heat pain threshold), the thermo-sensory limen (TSL) and paradoxical heat sensation (PHS) determined during TSL examination, mechanical and vibration detection threshold (MDT and CDT), mechanical and pressure pain threshold (MPT and PPT), mechanical pain sensitivity (MPS) and dynamic mechanic allodynia (DMA) as well as pain summation to repeated mechanical stimuli (WUR: wind-up ratio).

All sensory tests were performed at both dorsomedial feet. To compare individual QST data of patients with age and gender-matched control reference data from the DFNS^37^, raw data was *z*-transformed (except for PHS and DMA) by the software Equista® (Version 1.2.2, Statconsult, Magdeburg, Germany) using the following calculation:

$$z{-}value = \frac{mean\;individual\;value - mean\;reference\;data}{{SD\;of\;reference\;data}}.$$

Per definition, *z*-scores of zero represent a value corresponding to the mean of the healthy control cohort, positive *z*-scores indicate a gain of function, i.e. the patient was more sensitive to the test stimuli compared with controls (hyperaesthesia or hyperalgesia), whereas negative *z*-scores indicate a loss of function referring to a lower sensitivity of the patient (hypoaesthesia or hypoalgesia). A value outside the 95%-confidence interval of published data from healthy subjects (corresponding to *z*-values higher than + 1.96 or lower than − 1.96) is considered abnormal^36,37^.

### Statistical analysis

Statistical analyses were performed with custom S scripts written for the environment R 4.2.3 on macOS^38^. Depending on the class of analysed data and possible direction of causality, distributions were compared with linear regression, 2-sample Student’s *t*-test (continuous variables) or χ^2^ test (counts). Three groups with low, intermediate and high antibody titres, respectively, were compared with one-way ANOVA and post-hoc Tukey's honest significance test to control for type 1 error rate in the setting of multiple testing.

For autonomic function, sample size calculations and power analysis were performed with the R package pwr for RMSSD as primary outcome measure. A priori estimates were based on empirical distributions for RMSSD and the assumption that a drop by one-third would be relevant^39^. Under these assumptions, a sample size of 10 per group is necessary to achieve a power of 0.8 in the comparison of three zones of antibody titres. A sample size of about 40 is necessary for group comparison between autoimmune thyroiditis and healthy controls (Supplementary Fig. 1).

Using DFNS-QST, since all values are z-transformed, testing for deviations from normative values is based on z-tests. As a battery of 13 non-independent tests is performed, many assumptions of standard power calculations are infringed, and any power calculation, therefore, must be treated as indicative only. However, based on assuming a deviation of a z-value of ± 1, a group size of n = 17 is needed to achieve a power of 80% at an alpha-level of *p* < 0.05.

Where not otherwise specified, data are presented as mean value ± standard error of the mean (SEM) for continuous data or as absolute numbers (percentages) for count data. A *p*-value < 0.05 was considered statistically significant. Additionally, QST *z*-values were compared to the expected values of a healthy population with a mean *z*-value = 0 and a standard deviation = 1 using a *t*-test (*p* < 0.05). The percentage of abnormal QST values was additionally tested with χ^2^ tests for each of the 13 sensory parameters for sensory gain and 11 parameters for sensory loss and compared to a theoretically assumed frequency of 2.5% for each loss and gain, resulting from the definition of abnormal values as being outside the 95% CI of healthy controls.

## Results

### Patient characteristics

The basic clinical characteristics of the study population are reported in Table 1. There was no difference in anthropometric measures and common risk indicators for neuropathy, including Hb_A1c_ and vitamin B_12_ concentration, between the groups.

**Table 1:** Clinical characteristics of the study population (mean ± SEM or counts).

	Healthy controls (n = 27)	Subjects with autoimmune thyroiditis (n = 29)	*p*
Age, years	38 ± 4.3	47 ± 2.8	n. s
Sex			
Female	20	27	n. s
Male	7	2	
Body mass index, kg/m^2^	23.7 ± 0.6	25.6 ± 0.8	n. s
Hb_A1c_, %	5.3 ± 0.1	5.4 ± 0.1	n. s
Vitamin B_12_, ng/L	370 ± 58	389 ± 65	n. s
L-T4 dosage {µg per day)	0 ± 0	72 ± 9	< 1e−7

#### Parameters of thyroid function and autoimmunity

Although TSH and FT3 concentrations were identical in both groups, FT4 concentration was higher within the reference range and SPINA-GD was lower in the AIT group (Table 2). In the ultrasonographic evaluation, hypoechogenic or inhomogeneous patterns of thyroid parenchyma were observed in the majority of patients, and TPOAb titres were significantly higher in the group with AIT.

**Table 2:** Functional thyroid parameters in patients with AIT and controls.

Parameter (reference range)	Healthy controls (n = 27)	Subjects with autoimmune thyroiditis (n = 28)	*p*
TSH (0.3–0.35 mIU/L)	1.45 ± 0.12	1.98 ± 0.49	n. s
FT4 (9–26 pmol/L)	10.2 ± 0.3	12.7 ± 0.5	< 1e−4
FT3 (3.5–6.3 pmol/L)	5.2 ± 0.1	4.9 ± 0.1	n. s
SPINA-GT (1.41–8.67 pmol/s)	2.61 ± 0.26	N/A	N/A
SPINA-GD (20–60 nmol/s)	47.8 ± 1.1	37.3 ± 1.6	< 1e−5
JTI (1.3–4.1)	1.6 ± 0.1	1.8 ± 0.2	n. s
Thyroid volume, mL	11.3 ± 1.4	9.9 ± 1.9	n. s
Hypoechogenic pattern	0 (0%)	15 (54%)	< 0.01
Inhomogeneous pattern	0 (0%)	26 (93%)	< 1e−6
anti-Tg Ab (< 60 U/mL)	22.8 ± 6.1	243.2 ± 158.6	n. s
anti-TPO Ab (< 60 U/mL)	31.3 ± 9.8	1328.0 ± 302.9	< 0.001
TRAb (< 2 IU/L)	0.54 ± 0.04	0.52 ± 0.02	n. s

### Quantitative sensory testing

**Figure 1:**
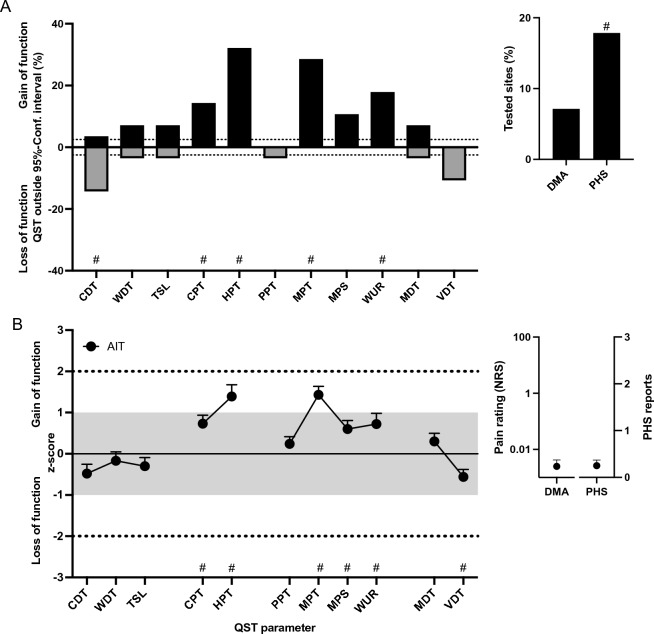
Frequencies of abnormal QST findings (**A**) and mean sensory profiles (**B**) in patients with treated AIT. (**A**) Each column gives the percentage of patients with abnormal findings for a particular quantitative sensory testing (QST) parameter (outside the 95% confidence interval [CI] of healthy controls). Positive values indicate positive sensory signs (hyperalgesia); negative values indicate negative sensory signs (hypoesthesia, hypoalgesia). Dashed lines show expected value for healthy controls (± 2.5%). ^#^*p* < 0.05 compared to the expected value of a healthy population. (**B**) Sensory profiles of all AIT patients presented as mean z scores + 95% CI. Positive z scores indicate positive sensory signs (hyperalgesia); negative z values indicate negative sensory signs (hypoaesthesia, hypoalgesia). Dashed lines show 95% CI for healthy controls (− 1.96 < z < 1.96). ^#^QST z-values were abnormal compared to the expected values of an ideal healthy population with a mean z-value = 0 and a standard deviation = 1. Data are presented in Mean + SEM. CDT = cold detection threshold; CPT = cold pain threshold; HPT = heat pain threshold; MDT = mechanical detection threshold; MPS = mechanical pain sensitivity; MPT = mechanical pain threshold; PPT = pressure pain threshold; TSL = thermal sensory limen; VDT = vibration detection threshold; WDT = warm detection threshold; WUR = wind-up ratio.

Of all included individuals 28 with AIT underwent QST. Comparing the AIT subjects to the expected values of a healthy population according to the reference data of the DFNS (mean ± SD: 0 ± 1 *z*-values^37^) the frequency of abnormal values for cold detection threshold (CDT), cold pain threshold (CPT), heat pain threshold (HPT), mechanical pain threshold (MPT), and wind-up ratio (WUR) were significantly higher. *Z*-values for cold pain threshold (CPT), heat pain threshold (HPT), mechanical pain threshold (MPT), and wind-up ratio (WUR) were higher and for vibration detection threshold (VDT) lower in AIT (Fig. 1B).

10 patients (36%) showed at least one abnormal decreased detection threshold indicating sensory loss of small (n = 7; 25%, 5 of these with PHS) or large fibre (n = 3) function (Fig. 1B).

16 other AIT patients (57%), with normal detection thresholds, showed at least one abnormal pain threshold. Heat hyperalgesia was the most abundant abnormal pain threshold displayed by 32% of all AIT patients (Fig. 1A) followed by cold hyperalgesia (14%).

Analysing the subgroups of patients with low, intermediate and high Anti-TPO antibody titres did not reveal a distinct somatosensory pattern or pathology. Anti-TPO antibody titre, TG antibody titre, TSH, FT4 concentration and SPINA-GD/-GT or JTI did not associate with any QST parameter, except for a significant positive correlation between SPINA-GT and mechanical pain sensitivity (MPS, r = 0.70, *p* = 0.014) and between FT3 levels and the mechanical detection threshold (MDT, r = 0.465, *p* = 0.013). Scores for anxiety, depression and quality of life did not correlate to any of the QST parameters.

### Cardiovascular autonomic function and thyroid disease

With the exception of a significantly higher drop in systolic blood pressure after orthostatic load in subjects with AIT, markers of autonomic function did not differ between the overall groups of patients and healthy controls (Supplementary Table 2). In some of the subtests, especially the frequency domain of resting condition, abnormal results were also found in the low zone of autoimmunity (Supplementary Table 3). However, there was a significant association between several markers of respiratory arrhythmia with titres of anti-TPO antibodies and a concentration-dependent relationship between the drop of systolic blood pressure and antibody titres (Tables 3 and 4). In persons not on L-T4 therapy SPINA-GT significantly correlated to the coefficient of variation of NN intervals in deep respiration (Supplementary Fig. 3).

**Table 3:** Cardiovascular autonomic function in three zones of autoimmunity defined by anti TPO antibody titres.

Parameter	Low (< 60 U/mL, n = 31)	Intermediate (60–1000 U/mL, n = 12)	High (> 1000 U/mL, n = 12)	*P* for trend
Resting condition				
Heart rate (bpm)	67.9 ± 1.9	62.7 ± 1.5	62.8 ± 3.6	n. s
SD NN (ms)	52.4 ± 2.9	48.9 ± 7.2	47.0 ± 7.4	n. s
CoV NN (%)	6.0 ± 0.4	5.0 ± 0.7	4.8 ± 0.6	n. s
RMSSD (ms)	40.9 ± 2.3	41.3 ± 8.2	40.9 ± 9.3	n. s
HRV index	12.4 ± 0.7	12.7 ± 1.3	11.5 ± 1.3	n. s
TINN (ms)	236.3 ± 10.2	208.5 ± 31.7	228.8 ± 23.9	n. s
VLF power (ms^2^)	5.3 ± 1.2	2.6 ± 1.0	2.5 ± .06	n. s
LF power (ms^2^)	5.0 ± 1.1	2.3 ± 1.0	2.1 ± 0.4	n. s
HF power (ms^2^)	5.1 ± 1.3	2.1 ± 0.7	2.0 ± 0.6	n. s
Gerritsen’s ratio	0.48 ± 0.03	0.50 ± 0.05	0.51 ± 0.06	n. s
Deep respiration				
SD NN (ms)	96.9 ± 7.4	76.0 ± 11.1	52.3 ± 5.5*	< 0.05
CoV NN (%)	11.4 ± 0.9	8.8 ± 1.4	6.4 ± 0.8*	< 0.05
RMSSD (ms)	54.1 ± 3.9	42.2 ± 5.4	28.9 ± 2.3*	< 0.05
MCR	0.06 ± 0.01	0.04 ± 0.01	0.03 ± 0.00	n. s
HRV index	14.4 ± 0.9	13.7 ± 1.4	10.7 ± 1.3	n. s
TINN (ms)	139.0 ± 13.2	157.1 ± 18.0	201.6 ± 19.4	n. s
Mean R-R_max_–R- R_min_	461.0 ± 47.5	321.9 ± 47.0	231.4 ± 27.8	< 0.05
Mean R-R_max_/R- R_min_	2.5 ± 0.4	1.6 ± 0.1	1.4 ± 0.1	n. s
Ewing's orthostasis test				
Mean R-R_max_/R-R_min_	1.5 ± 0.1	1.5 ± 0.1	1.3 ± 0.1	n. s
HRV index	9.6 ± 0.4	8.6 ± 0.9	8.3 ± 0.6	n. s
TINN (ms)	82.0 ± 6.6	73.8 ± 12.0	88.7 ± 11.7	n. s
Drop in systolic bp (mmHg)	5.5 ± 2.0	10.0 ± 3–3	22.6 ± 4.7***	< 0.001

p* < 0.05 *** < 0.001 compared to group with low antibody titres (< 60 U/mL). See Supplementary Fig. 2 for individual participant data of significant results.

**Table 4:** Cardiovascular autonomic function in controls and patients with AIT and medium and high anti-TPO antibody titres.

Parameter	Healthy controls (n = 25)	Intermediate (60–1000 U/mL, n = 12)	High (> 1000 U/mL, n = 12)	*P* for trend
Resting condition				
Heart rate (bpm)	67.4 ± 2.1	62.6 ± 1.5	62.8 ± 3.6	n. s
SD NN (ms)	52.7 ± 2.3	48.9 ± 7.2	47.0 ± 7.4	n. s
CoV NN (%)	6.0 ± 0.4	5.0 ± 0.7	4.8 ± 0.6	n. s
RMSSD (ms)	41.3 ± 2.5	41.3 ± 8.2	40.9 ± 9.3	n. s
HRV index	12.3 ± 0.7	12.7 ± 1.3	11.5 ± 1.3	n. s
TINN (ms)	237.6 ± 10.6	208.5 ± 31.7	228.8 ± 23.9	n. s
VLF power (ms^2^)	5.23 ± 1.38	2.60 ± 0.99	2.46 ± 0.58	n. s
LF power (ms^2^)	5.15 ± 1.31	2.30 ± 1.03	2.07 ± 0.44	n. s
HF power (ms^2^)	5.25 ± 1.44	2.08 ± 0.73	2.03 ± 0.64	n. s
Gerritsen’s ratio	0.47 ± 0.03	0.50 ± 0.05	0.51 ± 0.07	n. s
Deep respiration				
SD NN (ms)	89.00 ± 6.96	75.96 ± 11.13	52.33 ± 5.50	n. s
CoV NN (%)	10.52 ± 0.86	8.77 ± 1.37	6.41 ± 0.81	n. s
RMSSD (ms)	52.47 ± 4.44	42.20 ± 5.36	28.85 ± 2.31*	< 0.05
MCR	0.05 ± 0.01	0.04 ± 0.01	0.03 ± 0.00	n. s
HRV index	13.92 ± 1.05	13.68 ± 1.38	10.66 ± 1.33	n. s
TINN (ms)	143.60 ± 14.17	147.08 ± 18.04	201.56 ± 19.36	n. s
Mean R-R_max_–R-R_min_	448.24 ± 46.49	321.89 ± 47.04	231.40 ± 27.79	< 0.05
Mean R-R_max_/R-R_min_	2.16 ± 0.30	1.57 ± 0.15	1.41 ± 0.13	n. s
Ewing's orthostasis test				
Mean R-R_max_/R-R_min_	1.51 ± 0.09	1.45 ± 0.15	1.33 ± 0.10	n. s
HRV index	9.74 ± 0.47	8.58 ± 0.88	8.31 ± 0.58	n. s
TINN (ms)	82.36 ± 7.51	73.78 ± 12.01	88.87 ± 11.71	n. s
Drop in systolic bp (mmHg)	2.96 ± 1.80	10.00 ± 3.33	22.56 ± 4.67***†	< 0.001

p* < 0.05 *** < 0.001 compared to the group with low antibody titres (< 60 U/mL); † *p* < 0.05 compared to the group with intermediate antibody titres (60–1000 U/mL).

### Anxiety, depression and quality of life

**Figure 2:**
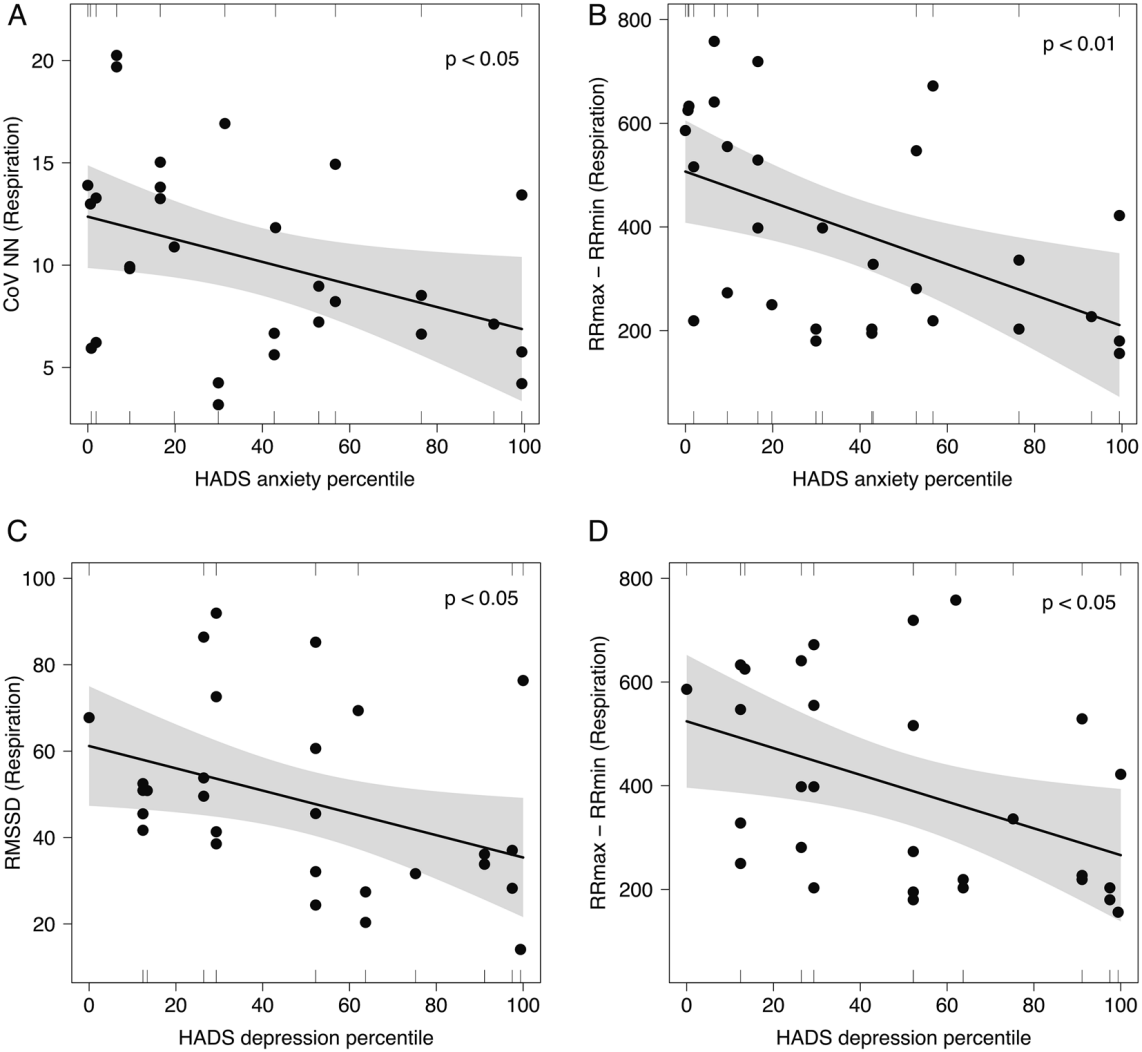
Correlation of respiratory arrhythmia to anxiety (**A**, **B**) and depression (**C**, **D**).

Anxiety and depression did not correlate to TSH, free T4 or free T3 concentrations, nor to SPINA-GT or markers for the central set point of thyroid homeostasis. However, both anxiety and depression were significantly more expressed in subjects with AIT (Supplementary Table 4), and they were also associated with antibody titres (Table 5). Additionally, both anxiety and depression percentiles correlated to certain markers of respiratory arrhythmia (Fig. 2), and depression was negatively correlated to SPINA-GD (adjusted R^2^ = 0.22, *p* < 0.01, Supplementary Fig. 3).

**Table 5:** Anxiety and depression in three zones of autoimmunity.

Result	Low (< 60 U/mL, n = 31, 19 replied to questions)	Intermediate (60–1000 U/mL, n = 12, 8 replied to questions)	High (> 1000 U/mL, n = 12, 7 replied to questions)	*P* for trend
Positive two-question test	0 (0%)	2 (25%)	2 (29%)	n. s
HADS anxiety percentile	26.3 ± 4.7	45.5 ± 13.0	65.3 ± 13.4**	< 0.01
HADS depression percentile	27.6 ± 3.5	79.2 ± 7.1*****	70.1 ± 10.7****	< 1e‒6

p** < 0.01, **** < 1e−4, ***** < 1e−5 compared to group with low antibody titres (anti-TPO Ab < 60 U/mL).

In the SF-36 questionnaire, summative physical functioning, social role functioning, emotional role functioning, vitality and mental health all correlated positively to SPINA-GD (*p* < 0.05, and < 0.01 for mental health, adjusted R^2^ = 0.10, 0.11, 0.08, 0.11 and 0.18, respectively). Likewise, mean R-R_max_–R-R_min_ in deep respiration correlated to emotional role functioning, vitality and mental health in the SF-36 survey (*p* < 0.05 and R^2^ = 0.11, 0.11 and 0.16, respectively).All subscales of the SF-36 questionnaire were reduced in AIT compared to controls (Supplementary Fig. 4).

## Discussion

In this study, we analysed a potential association between thyroid function, thyroid autoimmunity, somatosensory and autonomic nerve function, as well as the quality of life in patients with autoimmune thyroiditis and healthy control subjects. The main findings were: (1) detection thresholds assessed by QST were mostly within the normal range in AIT patients; however, a subgroup of 36% showed signs of small or large fibre dysfunction indicating a somatosensory loss; (2) up to one third of the AIT patients presented with thermal or mechanical hyperalgesia, indicating sensitization phenomena; (3) abnormal test results in the analysis of heart rate variability were common in both AIT and healthy controls, although the overall heart rate variability is similar in both groups; (4) markers of respiratory arrhythmia are significantly lower in subjects with high TPOAb titres, and most of them decrease in a manner that depends on antibody concentration; (5) anxiety, depression and reduced quality of life were more profoundly expressed in subjects with AIT and high antibody titres, and they correlated to HRV in deep respiration.

Our observations confirm earlier observations of impaired QoL in both autoimmune thyroiditis^40^, treated hypothyroidism^41^ and small fibre neuropathy^42^ and of reduced heart rate variability as a risk factor for depression^43^. Syndrome T or SORSHOT, i.e., continued symptoms and reduced quality of life in treated hypothyroidism and AIT continues to be a challenge in about 10% of patients with thyroid diseases^2,9,26,44–46^. Multiple hypotheses have been proposed for an explanation of this common, yet poorly understood condition, but up to now no satisfactory solution is available^47–51^. Therefore, research programs addressing potential pathophysiological mechanisms of syndrome T are highly needed.

Among the potential mechanisms that could underlie syndrome T are autonomic dysfunction and peripheral neuropathy, which were described extensively for untreated overt and subclinical hypothyroidism^3,13,14,52–56^. Dysfunction of small fibres in early autoimmune thyroiditis was reversible by replacement therapy achieving a biochemically euthyroid state^18,57^. However, complete remission of peripheral nerve function due to treatment with levothyroxine remains questionable. Ørstavik et al. investigated 38 patients with treated hypothyroidism and painful extremities and found signs of both large fibre neuropathy (assessed by nerve conduction studies) and small fibre neuropathy (based on increased thermal detection threshold) as well as central sensitization in a subgroup of patients^1^. Another study found, besides abnormal nerve conduction and QST abnormalities, also reduced intraepidermal nerve fibre density. However, in most of the included patients, other causes of neuropathy were identified, e.g., impaired glucose tolerance^4^.

In our study, no patients with pain in the extremities or competing reasons for polyneuropathy were included. Our study shows that in a subgroup after all about one-third of the patients without any other relevant comorbidity have distinct indications of somatosensory nerve dysfunction: 36% showed at least one abnormal decreased detection threshold indicating small (n = 7.25%, 5 of these with PHS) or large fibre (n = 3) neuropathy (Fig. 1B). Predominantly it concerns disturbances of the small nerve fibres that manifest as cold hypoaesthesia and PHS. PHS can indicate a loss of Aδ fibres and is in mild cases often the first sign of an incipient polyneuropathy, as previously described in children and adults with diabetes mellitus^58,59^. Large fibre neuropathies are rare, and a routine examination only with the vibration fork would not reveal the neuropathy. 57% of patients with normal detection thresholds showed at least one abnormal pain threshold predominantly heat hyperalgesia, that can indicate peripheral sensitization.

Of course, these results based only on QST must still be interpreted with caution. QST is a psychophysiological approach requiring the active cooperation of the subject, but due to the highly standardized technique in an experienced laboratory and the availability of a large database, no abnormal values above 5% are to be expected for the individual QST parameters in healthy subjects. QST has the advantage that it can detect both large and small fibre dysfunction, however it does not allow a differentiation between a peripheral or central origin of the impaired function. Indeed, the actual gold standard for detection of small fibre neuropathy would be a reduced intraepidermal nerve fibre density in the skin biopsy. Therefore, we cannot derive from the results the exact origin of the somatosensory dysfunction. Nevertheless, in the context of peripheral neuropathy in patients with hypothyroidism, based on nerve conduction studies and skin biopsies^4,15^, also in our cohort a peripheral origin of the somatosensory disturbances might be hypothesised.

Certainly, more difficulties for interpretation arise from the high rate of hyperalgesia. The pathomechanism remains unclear. Possible mechanisms include inflammation as well as down-regulation of potassium channels^60^ due to hypothyroidism, oxidative stress and glial cell apoptosis via TSH signalling^61^ and modulation of the TRPV1 and TRPM8 receptor properties by endogenous thyroid hormone metabolites^62–65^.

The QST data suggest that in a subgroup of treated AIT symptoms of mild somatosensory loss and heat hyperalgesia as a sign of peripheral sensitization may contribute to the reduced quality of life in affected persons. Unlike heat hyperalgesia, the high prevalence of mechanical hyperalgesia (29% in AIT subjects) might at least partly result from a slight modification in the QST instructions after the establishment of the reference database and does not definitely reflect an actual pathology. Associations with antibody titres could not be found in the present study. In summary, there is an overall faint association between peripheral nerve fibre function and syndrome T in a cohort, where other risk factors for peripheral neuropathy have been excluded.

In contrast, we observed a more pronounced association for functional parameters of the autonomic nervous system. Several studies and animal experiments found altered cardiovascular autonomic function in subclinical or overt hypothyroidism^53–56,66–70^. However, the evidence for a potential role of autoimmunity in euthyroid subjects is much weaker. A few studies and case reports suggested that such an association may exist, but the sample sizes were very small or autonomic function was only evaluated with indirect biomarkers^71–73^.

In our cohort, we found a significant and concentration-dependent association of impaired respiratory arrhythmia with thyroid autoimmunity. Both anti-TPO antibody titres and autonomic function correlated to scales of anxiety, depression and quality of life as well. With the exception of L-T4 substitution dosage and SPINA-GD, which both correlated to depression percentiles in HADS, no association between depression and thyroid function could be identified. Likewise, we did not find an association of autonomic function with parameters of thyroid homeostasis (except for a correlation between respiratory arrhythmia and SPINA-GT, a parameter that can, however, not be sensibly calculated in persons on L-T4). This result is in contrast to earlier studies that suggested a strong association of sensorimotor and autonomic function with both hypothyroidism^3,13,14,16^ and thyrotoxicosis^14,74,75^. The lack of a strong association with thyroid function in our study probably results from the fact that we included biochemically euthyroid persons receiving sufficient substitution therapy only. Due to the therapeutic fixation of thyroid function in the euthyroid zone, potential effects of thyroid hormones on the nervous system are largely concealed.

Therefore, our results do not give rise to the conclusion that thyroid homeostasis plays no or a minor role in sensorimotor or autonomic function. They suggest, however, a certain (and probably additional) effect of thyroid autoimmunity on the pathogenesis of at least the neurological and mental phenotypes of syndrome T. Furthermore, they could explain why certain immune-modulatory and neurotropic treatment options, e.g., with selenium or vitamin D substitution, may be beneficial in a subgroup of affected subjects^76,77^. If confirmed by additional studies these observations might pave the way for future personalised therapeutic approaches.

Strengths of our study include the comprehensive investigation of peripheral and autonomic nerve function, the comparability of both AIT and control group and the careful exclusion of important confounders leading to neuropathy, including diabetes mellitus, alcohol abuse, systemic rheumatic diseases and chemotherapy with platinum-based substances. Weaknesses apply mainly to the low sample size. Several HRV results in Ewing’s orthostasis test were insignificant with comparably low p values between 0.05 and 0.10 and a potential antibody concentration-dependent trend. These results could be clearer with a larger sample size.

The number of included persons provides sufficient power for the analysis of the association between thyroid immunity and heart rate variability in deep respiration (Supplementary Fig. 1). However, addressing the question if autoimmune thyroiditis per se (i. e. irrespective of the extent of autoimmunity) could contribute to peripheral nerve dysfunction would require a very large study. The results of this small study could provide the basis for sufficiently powered confirmation studies that are needed to validate our findings and provide more evidence on the role of thyroid autoimmunity.

Since patients with AIT were recruited from a tertiary university-affiliated endocrine centre, no reliable figures for the prevalence of neural dysfunction in treated thyroid disease can be provided. Elucidating this question would require a large field study including subjects recruited from primary physicians or a population-based sample.

We found a three-way association between psychological aspects (including anxiety, depression and self-reported quality of life), antibody expression and autonomic function. Of course, neither causation nor the direction of potential causality can be revealed in an observational study. Since a truly experimental approach is not feasible this may be partly remedied by modern statistical methods, e.g., instrument variable regression. This approach would require, however, considerably larger sample sizes.

## Conclusion

Autoimmune thyroiditis may involve autonomic dysfunction, even in subjects receiving adequate substitutive therapy with levothyroxine. In treated hypothyroidism, cardiovascular autonomic function is predicted by thyroid autoimmunity rather than thyroid function. One-third of the examined AIT patients exhibit at least one abnormal decreased detection threshold, mostly in the form of paradoxical heat sensation as a sign of small fibre dysfunction. In half of the patients, at least one pain threshold (mostly for heat) was mildly decreased indicating peripheral sensitization.

### Supplementary Information

Supplementary Information 1.

Supplementary Information 2.

## Data Availability

HRV data of this study used for analysis are available in anonymized form (i.e., after removal of information that allows for identification of subjects) in the online supplement. Additional information is available at request from the corresponding author (JWD).
