# Supplementary material for: Impaired autonomic function and somatosensory disturbance in patients with treated autoimmune thyroiditis
Source: Sci Rep. 2024 May 29;14:12358. doi: 10.1038/s41598-024-63158-w (PMC11137073; doi:10.1038/s41598-024-63158-w)
Supplement: Supplementary file 2 — Supplementary Information 2. [file 41598_2024_63158_MOESM2_ESM.pdf]

scientific reports

Supplementary Material Accompanying the Publication "Impaired autonomic function and somatosensory disturbance in patients with treated autoimmune thyroiditis"

Bojana Bazika-Gerasch, Nina Kumowski, Elena Enax-Krumova, Miriam Kaisler, Lynn Bernadette Eitner, Christoph Maier, Johannes W. Dietrich*

***Correspondence:** Corresponding Author: johannes.dietrich@ruhr-uni-bochum.de

Calculations

SPINA-GT (thyroid's secretory capacity) was calculated with

$$\hat{G}_T = \frac{\beta_T (D_T + [TSH])(1 + K_{41}[TBG] + K_{42}[TTR])[FT_4]}{\alpha_T [TSH]}$$

from steady-state concentrations of TSH and FT4, and constant parameters for plasma protein binding, distribution and elimination, as previously described[1].

SPINA-GD (sum activity of step-up deiodinases) was calculated with

$$\hat{G}_D = \frac{\beta_{31}(K_{M1} + [FT_4])(1 + K_{30}[TBG])[FT_3]}{\alpha_{31}[FT_4]}$$

from steady-state concentrations of FT3, FT4 and predefined constant values for the kinetics of hormones[1].

Jostel's TSH index, an estimate for the central function of the feedback loop (the so-called set point) was calculated with[2]

$$TSHI = \ln([TSH]) + \beta[FT_4]$$

Supplementary Table 1: Parameters for calculation[1]

Parameter	Explanation	Value
α_T	Dilution factor for thyroxine	0.1 L^{-1}
β_T	Clearance exponent for T4	$1.1 \cdot 10^{-6}$ sec^{-1}
D_T	EC_{50} for TSH	2.75 mIU/L
K_{41}	Dissociation constant of T4 at thyroxine binding globulin	$2 \cdot 10^{10}$ L/mol
K_{42}	Dissociation constant of T4 at transthyretin	$2 \cdot 10^{8}$ L/mol
α_{31}	Dilution factor for triiodothyronine	0.026 l^{-1}
β_{31}	Clearance exponent for T3	$8 \cdot 10^{-6}$ sec^{-1}
K_{M1}	Association constant of type 1 deiodinase	500 nmol/L
K_{30}	Association constant of T3 at thyroxine-binding globulin	$2 \cdot 10^{9}$ L/mol
$[TBG]$	Standard concentration of thyroxine-binding globulin	300 nmol/L
$[TTR]$	Standard transthyretin concentration	4.5 µmol/L
β	Correction coefficient of logarithmic model	0.1345

Supplementary Figures

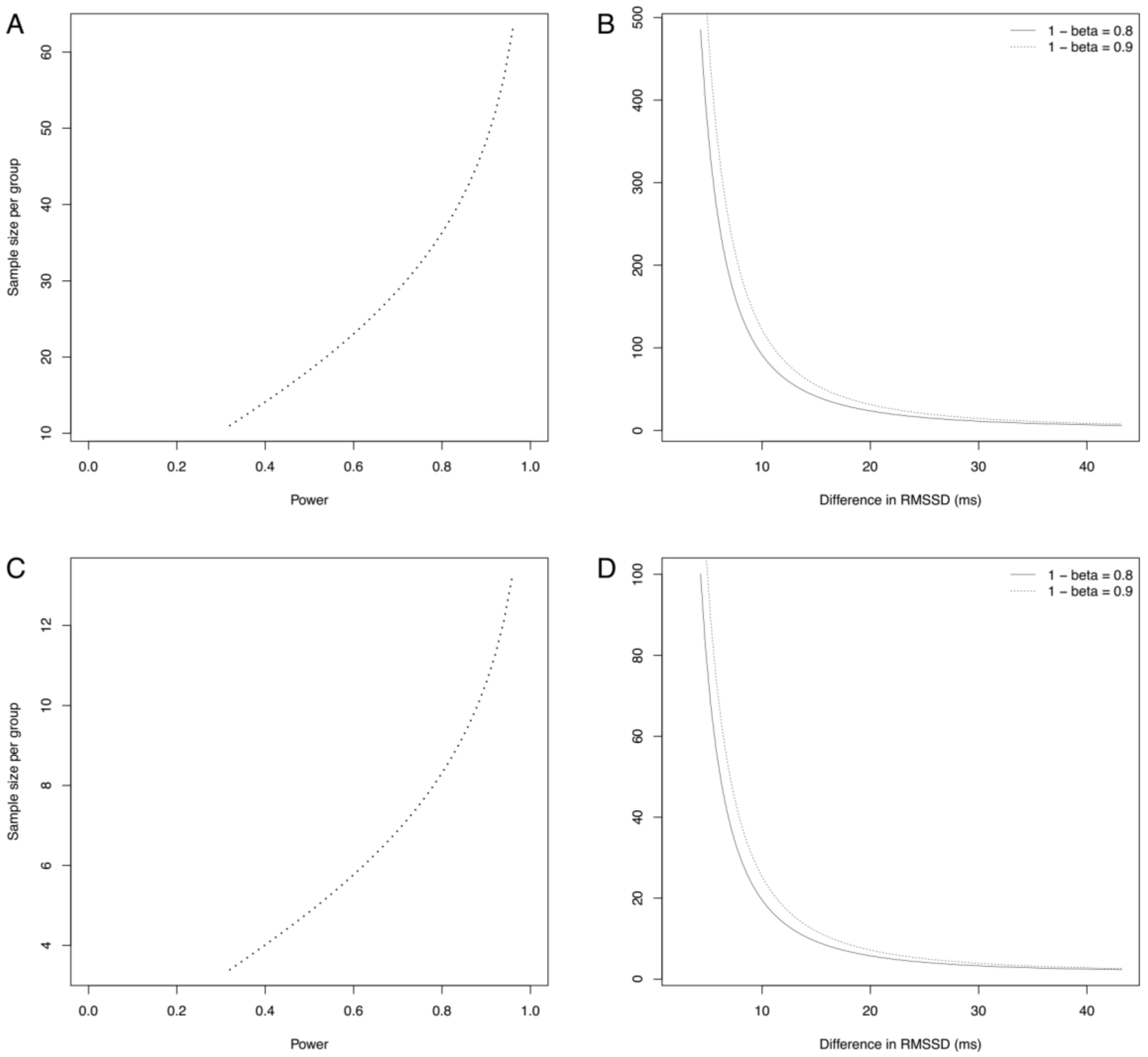

Supplementary Figure 1: Sample size depending on power for a drop in RMSSD by one-third (A, C) and depending on change in RMSSD (B, D). A and B refer to group comparison between autoimmune thyroiditis and healthy controls; C and D apply to comparing three zones of antibody titres.

Supplementary Results

Supplementary Table 2: Cardiovascular autonomic function in subjects with and without AIT

**Parameter**	**Healthy controls (n = 27)**	**Subjects with autoimmune thyroiditis (n = 28)**	**P**
Resting condition			
Heart rate (bpm)	67.3 ± 1.9	64.6 ± 2.1	n. s.
SD NN (ms)	50.9 ± 3.2	50.4 ± 4.2	n. s.
CoV NN (%)	5.7 ± 0.4	5.4 ± 0.4	n. s.
RMSSD (ms)	39.9 ± 2.5	41.8 ± 5.0	n. s.
HRV index	12.1 ± 0.7	12.6 ± 0.8	n. s.
TINN (ms)	231.7 ± 10.6	226.8 ± 17.7	n. s.
VLF power (ms^2)	4.9 ± 1.3	3.4 ± 0.7	n. s.
LF power (ms^2)	4.8 ± 1.2	2.9 ± 0.6	n. s.
HF power (ms^2)	4.9 ± 1.3	2.8 ± 0.6	n. s.
Gerritsen’s ratio	0.48 ± 0.03	0.50 ± 0.04	n. s.
Deep respiration			
SD NN (ms)	84.2 ± 7.1	86.0 ± 10.4	n. s.
CoV NN (%)	9.9 ± 0.9	10.2 ± 1.2	n. s.
RMSSD (ms)	50.5 ± 4.3	42.9 ± 4.5	n. s.
MCR	0.05 ± 0.01	0.05 ± 0.01	n. s.
HRV index	13.5 ± 1.0	13.9 ± 1.0	n. s.
TINN (ms)	144.0 ± 13.1	159.9 ± 15.3	n. s.
Mean $R\text{-}R_{max} – R\text{-}R_{min}$	425.9 ± 45.1	349.5 ± 54.0	n. s.
Mean $R\text{-}R_{max} / R\text{-}R_{min}$	2.1 ± 0.3	2.1 ± 0.4	n. s.
Ewing's orthostasis test			
Mean $R\text{-}R_{max} / R\text{-}R_{min}$	1.5 ± 0.1	1.3 ± 0.1	n. s.
HRV index	9.5 ± 0.5	8.7 ± 0.5	n. s.
TINN (ms)	82.3 ± 7.0	81.2 ± 7.4	n. s.
Drop in systolic bp (mmHg)	3.3 ± 1.7	16.9 ± 2.0	< 0.001

Supplementary Table 3: Proportion of abnormal results in three zones of autoimmunity defined by anti TPO antibody titres. Overt autonomic dysfunction: 2 or more subtests pathological. Orthostatic hypertension: Drop in systolic blood pressure of > 25 mmHg

**Condition / Subtest**	**Low (< 60 U/mL, n = 31)**	**Intermediate (60–1000 U/mL, n = 12)**	**High (> 1000 U/mL, n = 12)**	**P for trend**
Resting condition (time domain)	0 (0%)	0 (0%)	0 (0%)	n. s.
Resting condition (frequency domain)	4 (14.8%)	0 (0%)	0 (0%)	n. s.
Deep respiration	4 (16.7%)	4 (44.4%)	2 (40%)	n. s.
Ewing's orthostasis test	3 (11.1%)	0 (0%)	2 (25%)	n. s.
Overt autonomic dysfunction	1 (3.2%)	0 (0%)	1 (8.3%)	n. s.
Orthostatic hypertension	0 (0%)	0 (0%)	2 (22,2%)	< 0.05

Supplementary Table 4: Anxiety and depression in the two groups

**Result**	**Healthy controls (n = 27, 18 replied to questions)**	**Subjects with autoimmune thyroiditis (n = 28, 17 replied to questions)**	**P**
Positive two-question test	0 (0%)	5 (29%)	< 0.05
HADS anxiety percentile	27.2 ± 4.8	54.6 ± 9.1	< 0.05
HADS depression percentile	28.9 ± 3.9	72.1 ± 6.7	< 1e–5

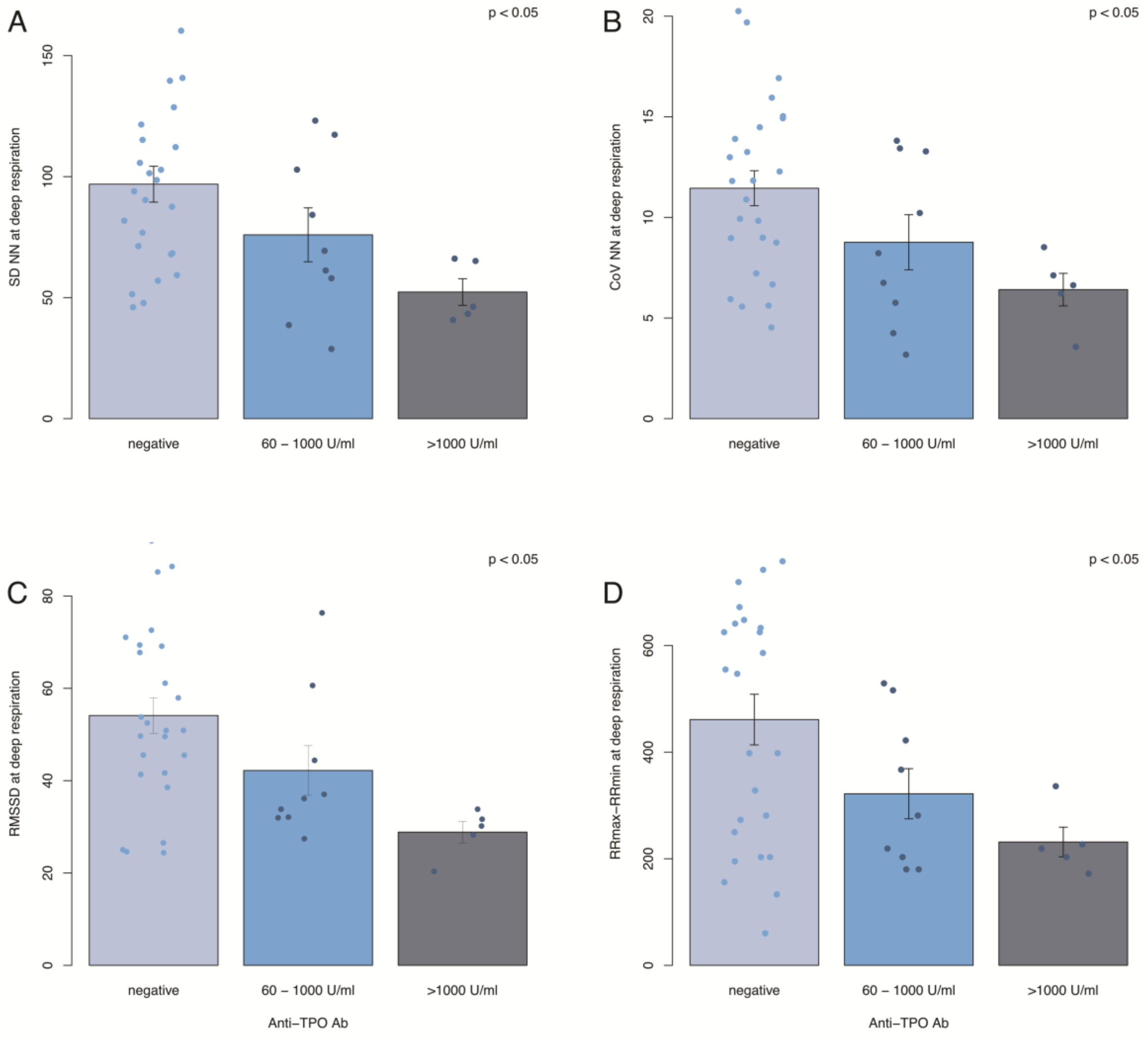

Supplementary Figure 2: Markers of respiratory arrhythmia in relation to anti-TPO antibody titres. Shown are means ± SEM (bars and whiskers) as well as results of individual participants (dots).

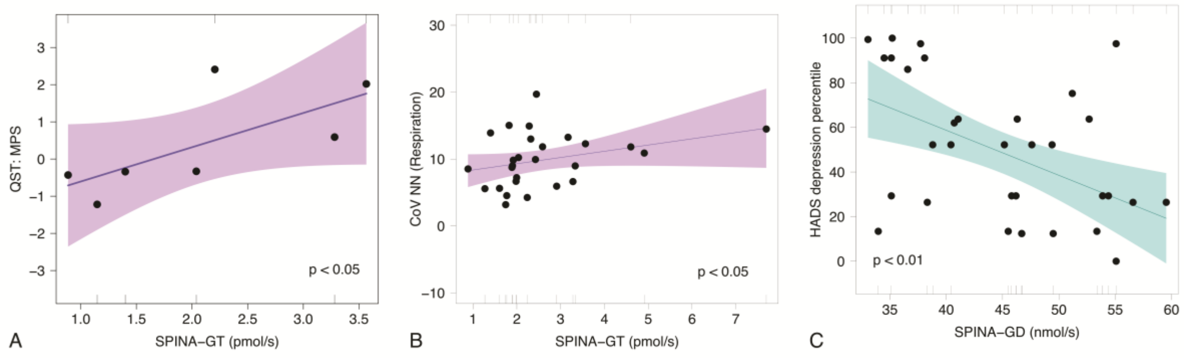

Supplementary Figure 3: Correlation of functional parameters of thyroid homeostasis with the mechanical pain sensitivity (MPS) of QST (A), respiratory arrhythmia in HRV analysis (B) and depression percentiles in HADS (C).

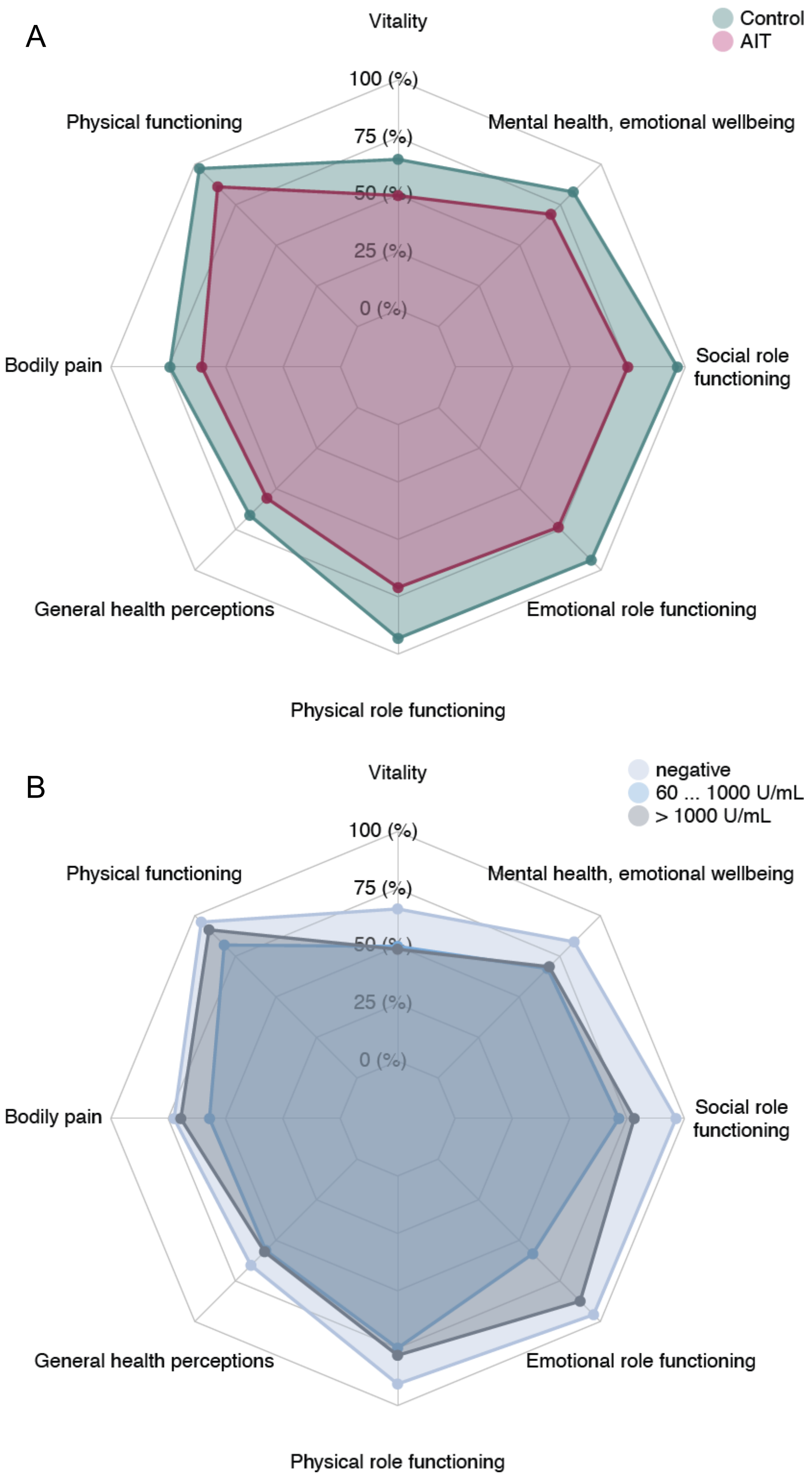

Supplementary Figure 4: Results of the SF-36 questionnaire in subjects with and without AIT (A) and with different levels of autoantibody titres (B).

Legends

Explanation of labels in primary data (as provided by the supplementary data table)

**Label**	**Explanation**
Dekade	Age decade (years)
Diagnose	Diagnosis (AIT = autoimmune thyroiditis; Gesund = healthy control)
Geschlecht	Sex (w = female; m = male)
Laenge	Body length (cm)
BMI	Body mass index (kg/m^2)
SubstDauer	Time on substitution therapy (years)
EuthDauer	Time euthyroid (years)
Dosis	L-T4 Dosage (µg per day)
SD.Volumen	Thyroid volume (ml)
echoarm	Hypoechogenic pattern in ultrasonography (0 = no; 1 = yes)
inhomogen	Inhomogeneous pattern in ultrasonography (0 = no; 1 = yes)
TSH	TSH concentration (mIU/L)
FT4	Free T4 concentration (ng/L)
FT3	Free T3 concentration (pmol/L)
TgAK	anti-Tg Ab (U/mL)
TPO.AK	anti-TPO Ab (U/mL)
TRAK	TSH receptor autoantibodies (IU/L)
HbA1c	HbA1c fraction (%)
VitB12	Vitamin B12 concentration (ng/L)
ProSciCard.HR_Ruhe	Heart rate in rest
ProSciCard.SD.RR_Ruhe	standard deviation of normal artefact-free RR intervals in rest
ProSciCard.VK_Ruhe	coefficient of variation of normal artefact-free RR intervals in rest
ProSciCard.RMSSD_Ruhe	root mean square of successive differences in rest
ProSciCard.HRV.Index_Ruhe	HRV triangular index in rest
ProSciCard.TINN_Ruhe	baseline width of the RR interval histogram in rest
ProSciCard.Ruhe.erfuellt	Normal HRV in rest? (ja = yes; nein = no)
ProSciCard.vaso	VLF band of power spectrum (VLF: 0.003 – 0.04 Hz)

ProSciCard.baro	LF band of power spectrum (0.04 – 0.15 Hz)
ProSciCard.resp	HF band of power spectrum (HF: 0.15 – 0.4 Hz)
Gerritsen	Gerritsen's ratio (LF / (LF + HF))
ProSciCard.Powerspektrum.erfuellt	Normal power spectrum in rest? (ja = yes; nein = no)
ProSciCard.HR_Atmung	Heart rate in deep respiration
ProSciCard.SD.RR_Atmung	standard deviation of normal artefact-free RR intervals in deep respiration
ProSciCard.VK_Atmung	coefficient of variation of normal artefact-free RR intervals in deep respiration
ProSciCard.RMSSD_Atmung	root mean square of successive differences in deep respiration
ProSciCard.R.Rmax.R.Rmin_Atmung	RR_{max}–RR_{min} in deep respiration
ProSciCard.R.Rmax.R.Rmin_Atmung.1	RR_{max}/RR_{min} in deep respiration
ProSciCard.MCR_Atmung	mean circular resultant in deep respiration
ProSciCard.HRV.Index_Atmung	HRV triangular index in deep respiration
ProSciCard.TINN_Atmung	baseline width of the RR interval histogram in deep respiration
ProSciCard.Atmung.erfuellt	Normal HRV in deep respiration? (ja = yes; nein = no)
ProSciCard.R.Rmax.R.Rmin_Valsalva	RR_{max}/RR_{min} in Valsalva's manoeuvre
ProSciCard.HRV.Index_Valsalva	HRV triangular index in Valsalva's manoeuvre
ProSciCard.TINN_Valsalva	baseline width of the RR interval histogram in Valsalva's manoeuvre
ProSciCard.Valsalva.erfuellt	Normal HRV in Valsalva's manoeuvre? (ja = yes; nein = no)
ProSciCard.R.Rmax.R.Rmin_Ewing	RR_{max}/RR_{min} in Ewing's test
ProSciCard.HRV.Index_Ewing	HRV triangular index in Ewing's test
ProSciCard.TINN_Ewing	baseline width of the RR interval histogram in Ewing's test
Blutdruckdifferenz	Drop in blood pressure in Ewing's test
ProSciCard.Ewing.erfuellt	Normal HRV in Ewing's test? (ja = yes; nein = no)
ProSciCard.Zeitdomaene.in.Ruhe.normal.	Normal HRV in rest? (ja = yes; nein = no)
ProSciCard.Powerspektrum.in.Ruhe.normal.	Normal power spectrum in rest? (ja = yes; nein = no)
ProSciCard.Tiefe.Atmung.normal.	Normal HRV in deep respiration? (ja = yes; nein = no)
ProSciCard.Valsalva.normal.	Normal HRV in Valsalva's manoeuvre? (ja = yes; nein = no)
ProSciCard.Ewing.normal.	Normal HRV in Ewing's test? (ja = yes; nein = no)
ProSciCard.Anzahl.Pathologisch	Number of pathological subtests
HADS.A.Perzentil	Anxiety percentile in the Hospital Anxiety and Depression Scale
HADS.A.T.Wert	Anxiety t value in the Hospital Anxiety and Depression Scale
HADS.D.Perzentil	Depression percentile in the Hospital Anxiety and Depression Scale
HADS.D.T.Wert	Depression t value in the Hospital Anxiety and Depression Scale
TQ.S	At least one of the questions in the two questions test positive? (1 = yes; 0 = no)

SF.36.KOEFU	Physical functioning in the SF-36 questionnaire
SF.36.KOERO	Physical role functioning in the SF-36 questionnaire
SF.36.SCHM	Pain in the SF-36 questionnaire
SF.36.AGES	General health perception in the SF-36 questionnaire
SF.36.VITA	Vitality in the SF-36 questionnaire
SF.36.SOFU	Social role functioning in the SF-36 questionnaire
SF.36.EMRO	Emotional role functioning in the SF-36 questionnaire
SF.36.PSYC	Mental health / emotional wellbeing in the SF-36 questionnaire
ThyPro.GoitreScale	ThyPro: Goitre symptoms
ThyPro.HyperthyroidScale	ThyPro: Hyperthyroid symptoms scale
ThyPro.HpothyroidScale	ThyPro: Hypothyroid symptoms scale
ThyPro.EyeScale	ThyPro: Eye symptoms scale
ThyPro.TirednessScale	ThyPro: Tiredness scale
ThyPro.CognitiveScale	ThyPro: Cognition scale
ThyPro.AnxietyScale	ThyPro: Anxiety scale
ThyPro.DepressivitySale	ThyPro: Depressivity scale
ThyPro.EmotionalScale	ThyPro: Emotional susceptibility scale
ThyPro.SocialScale	ThyPro: Impaired social life scale
ThyPro.DailyLifeScale	ThyPro: Impaired daily life scale
ThyPro.SexScale	ThyPro: Impaired sex life scale
ThyPro.CosmeticScale	ThyPro: Cosmetic complaints scale
FT4.SI	Free T4 concentration (pmol/L)
SPINA.GD	Whole body sum activity of peripheral step-up deiodinases (SPINA-GD, nmol/s)
SPINA.GT	Thyroid's secretory capacity (SPINA-GD, pmol/s)
JTI	Jostel's TSH index
TFQI	Thyroid Feedback Quantile-based Index
Vitamin.B12	Vitamin B12 concentration (ng/L, curated)
Diagnose2	Diagnosis (AIT = autoimmune thyroiditis; Kontrolle = healthy control)
group	Group (AIT = autoimmune thyroiditis; Kontrolle = healthy control)
group.e	Group (English labels)
TPO.AK.class	anti-TPO Ab class
Tg.AK.class	anti-Tg Ab class
TgAK.curated	TSH receptor autoantibodies class
TPO.AK.curated	anti-TPO Ab (U/mL, curated)

TRAK.curated	TSH receptor autoantibodies (IU/L, curated)
Crit.fulfilled	Criteria for autoimmune thyroiditis fulfilled? (1 = yes; 0 = no)
CAN1	Criteria for beginning autonomic neuropathy fulfilled? (1 = yes; 0 = no)
CAN2	Criteria for manifest autonomic neuropathy fulfilled? (1 = yes; 0 = no)

Supplementary References

1 Dietrich, J. W. *et al.* Calculated Parameters of Thyroid Homeostasis: Emerging Tools for Differential Diagnosis and Clinical Research. *Frontiers in endocrinology* **7**, 57, doi:10.3389/fendo.2016.00057 (2016).

2 Jostel, A., Ryder, W. D. & Shalet, S. M. The use of thyroid function tests in the diagnosis of hypopituitarism: definition and evaluation of the TSH Index. *Clin Endocrinol (Oxf)* **71**, 529-534, doi:10.1111/j.1365-2265.2009.03534.x (2009).
